# Case Report: A rare triad of neoplasms: navigating synchronous accessory breast cancer, papillary thyroid carcinoma, and inflammatory nasal papilloma

**DOI:** 10.3389/fmed.2026.1841765

**Published:** 2026-06-01

**Authors:** Xiangning Zeng, Huang Huang, Yuzhong Hong, Qin Meng, Kaihua Ye, Lian Zhou, Huajun Li, Ying Chen, Jiawei Huang, Chuxia Feng

**Affiliations:** 1 Department of Thyroid and Breast Surgery, The Second People’s Hospital of Qinzhou, Qinzhou, Guangxi, China; 2 Guangxi Hospital of the First Affiliated Hospital of Sun Yat-sen University, Nanning, Guangxi, China; 3 Xincheng County People’s Hospital, Xincheng, Guangxi, China; 4 Department of Thoracic Surgery, Gaozhou People’s Hospital, Maoming, Guangdong, China

**Keywords:** accessory breast cancer, multidisciplinary management, nasal papilloma, papillary thyroid carcinoma, synchronous tumors

## Abstract

**Background:**

The differential diagnosis of an isolated axillary mass in a woman with normal breast imaging presents a significant clinical challenge, with accessory breast carcinoma being a critical yet often overlooked entity. The concurrent management of multiple primary tumors further complicates therapeutic decision-making.

**Case presentation:**

A 60-year-old woman presented with a 1-year history of a painless, enlarging left axillary mass. Initial imaging revealed a BI-RADS 5 lesion in the left axilla with suspicious lymph nodes, but no primary breast abnormality; subsequent breast MRI confirmed the absence of an orthotopic primary. Core needle biopsy confirmed grade II invasive ductal carcinoma of mammary origin. Subsequent evaluations incidentally identified a Bethesda V thyroid nodule and a benign nasal papilloma. Following six cycles of neoadjuvant TAC chemotherapy, she underwent sequential surgeries: prophylactic ipsilateral nipple-sparing subcutaneous mastectomy with axillary lymph node dissection, endoscopic nasal tumor resection, and total thyroidectomy with central compartment dissection. Final pathology confirmed primary accessory breast carcinoma (ypT3N2, Luminal B), papillary thyroid carcinoma (pT1aN1a), and squamous epithelial papilloma of the nasal cavity. Adjuvant treatment included locoregional radiotherapy, endocrine therapy (letrozole plus abemaciclib), and radioiodine ablation.

**Conclusion:**

This case underscores that primary accessory breast cancer must be a leading consideration in the differential diagnosis of an isolated axillary mass with negative standard breast imaging. It also illustrates a pragmatic, multidisciplinary strategy for the integrated management of synchronous primary tumors, guided by the principles of oncologic prioritization and sequenced intervention.

## Introduction

An isolated axillary mass in a woman with normal breast imaging presents a critical diagnostic challenge. Among the differentials, primary carcinoma arising in accessory breast tissue is a rare but decisive diagnosis that is frequently missed, risking inappropriate clinical management.

This case report details a patient in whom such an axillary mass heralded the presence of three synchronous neoplasms: an aggressive accessory breast carcinoma, a papillary thyroid carcinoma, and a nasal papilloma. We describe the diagnostic pathway that confirmed accessory breast cancer, and present the prioritized, sequential multidisciplinary strategy that successfully integrated care for these co-existing tumors, offering a practical framework for similar complex presentations.

## Case

**Figure 1:**
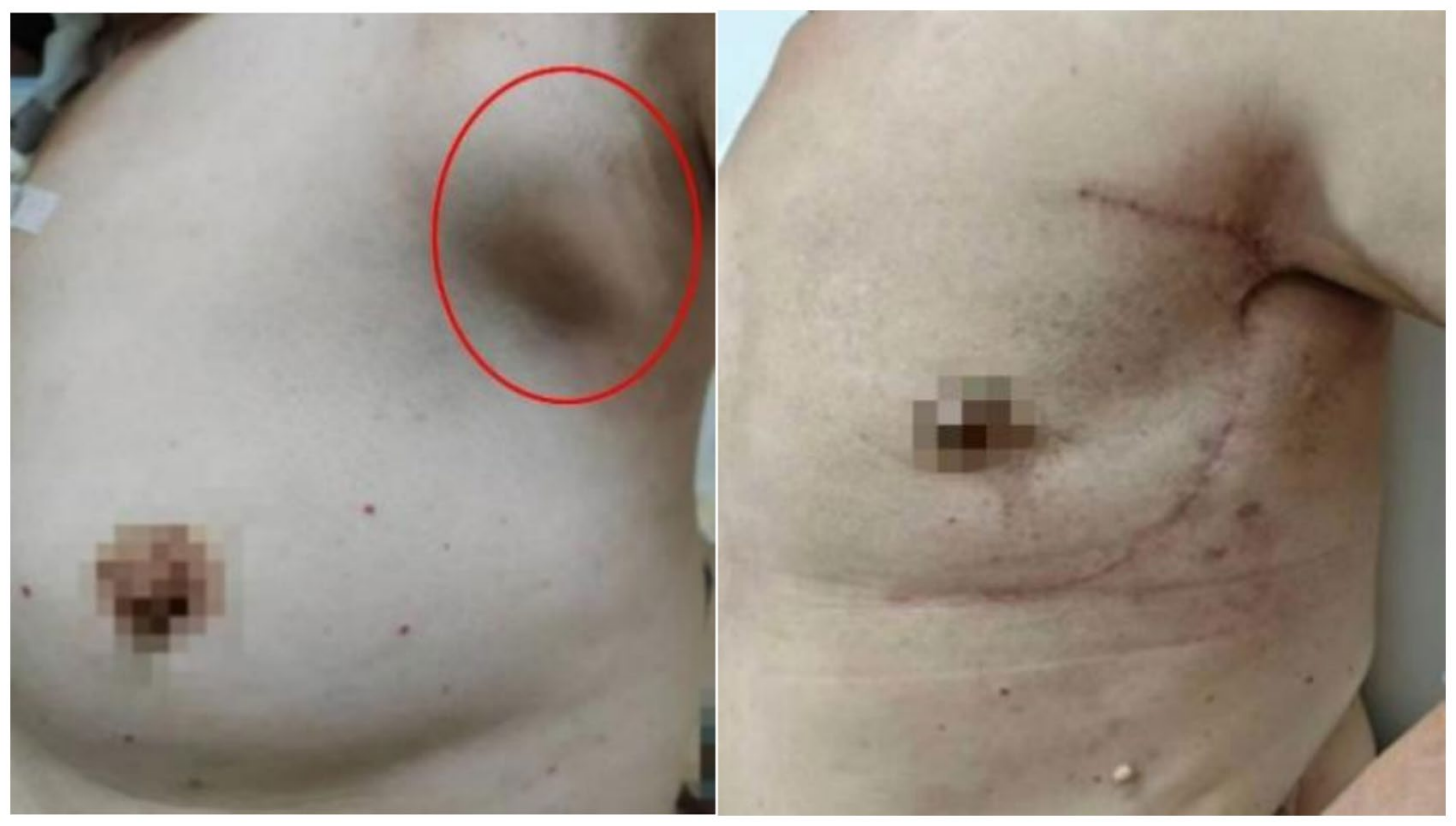
Comparison of left axillary mass at initial presentation and 8 months post-surgery.

**Figure 2:**
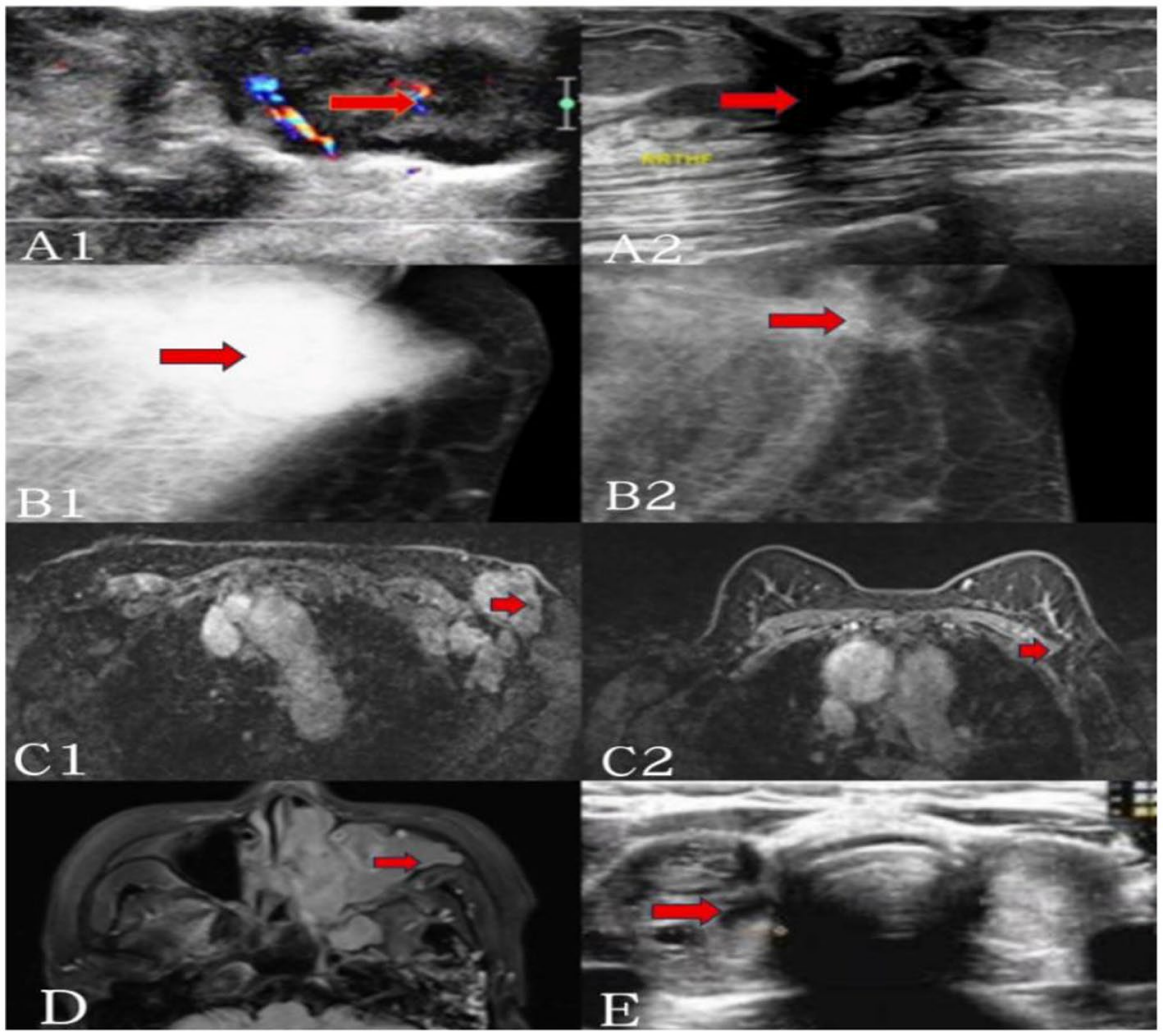
**(A1,A2)** Contrasting breast color Doppler ultrasound images of the left axillary mass before and after neoadjuvant therapy. **(B1,B2)** Contrasting mammography images of the left axillary mass before and after neoadjuvant therapy. **(C1,C2)** Contrasting magnetic resonance imaging of the left axillary mass before and after neoadjuvant therapy. **(D)** A nodular abnormal signal focus is observed in the left nasal cavity. It exhibits isointense signal on T1-weighted imaging (T1WI), slightly hyperintense to isointense mixed signal on T2-weighted imaging (T2WI), and slightly hyperintense signal on FLAIR. The margins are relatively well-defined, with dimensions approximately 61 mm × 24 mm × 19 mm. The lesion extends posteriorly into the nasopharyngeal cavity. **(E)** Pre-treatment color Doppler ultrasound of thyroid mass.

**Figure 3:**
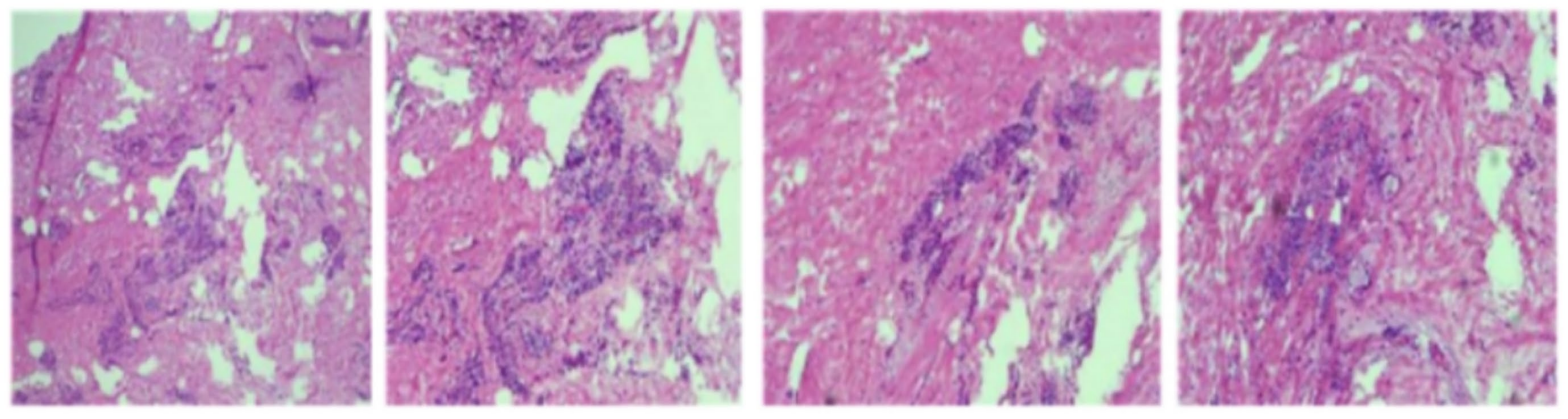
Postoperative pathology of left breast tumor (H&E × 400).

On 9 September 2024, a 60-year-old female patient was admitted to the Department of Thyroid and Breast Surgery at Qinzhou Second People’s Hospital. She presented with a progressively enlarging left axillary mass, which had been discovered over 1 year previously. Her past medical history included a bilateral partial thyroidectomy performed in 2011 for a benign thyroid tumor, a long-standing nasal cavity mass (present for >10 years), and hypertension of 4 years’ duration. Family history was significant for malignancy, with her husband having lung cancer and her daughter a teratoma. Physical examination revealed a firm, poorly mobile mass, approximately 5 cm in diameter, in the left axilla (Figure 1). No abnormalities were noted in the breasts or thyroid. Imaging investigations comprised a breast ultrasound, which identified a hypoechoic mass in the left axilla (54 × 35 × 33 mm; BI-RADS 5) accompanied by multiple enlarged lymph nodes (the largest measuring 18 × 12 mm), with no abnormalities detected in the breast parenchyma. Thyroid ultrasound showed cystic-solid nodules in both lobes (TI-RADS 3), with no abnormal cervical lymph nodes bilaterally. Subsequent breast MRI revealed no suspicious enhancement within the orthotopic breast parenchyma (1, 2). The admission diagnoses were: (1) left axillary mass, (2) left nasal cavity mass, (3) thyroid nodules, and (4) hypertension (Figures 2, 3).

**Figure 4:**
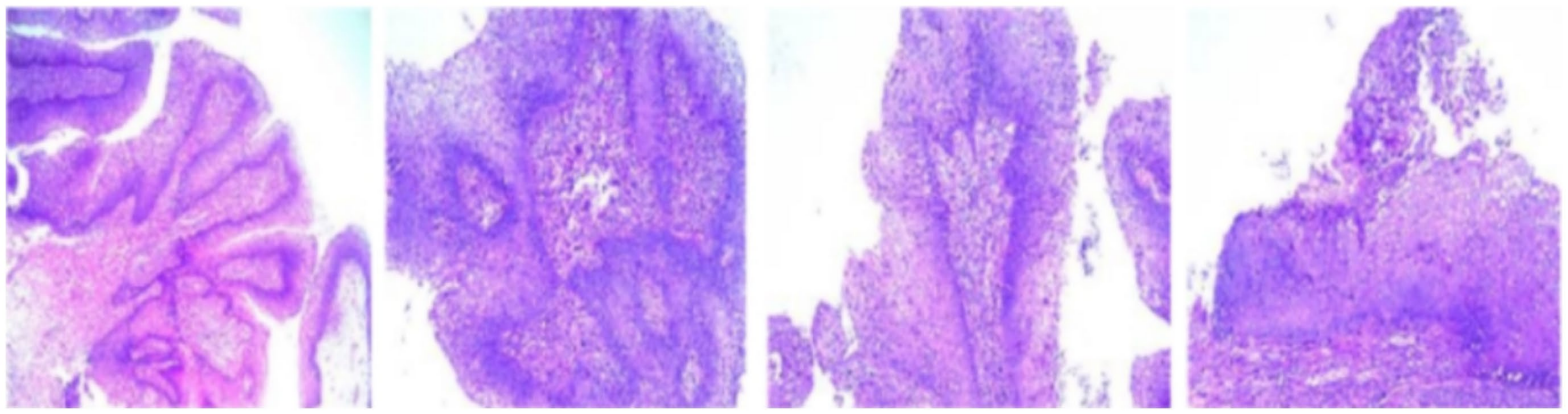
Postoperative pathology of nasal cavity tumor (H&E × 400).

The patient was diagnosed with multiple primary tumors, based on the distinct histopathological morphology of each lesion (ductal carcinoma in accessory breast tissue, papillary carcinoma of the thyroid, and nasal papilloma), their separate anatomical locations without locoregional continuity, and the exclusion of metastatic disease via immunohistochemical profiling and imaging. Following multiple multidisciplinary team (MDT) consultations, the process is detailed in Table 1.

**Table 1:** Treatment process.

Time	Site of disease	Diagnosis/treatment	Remarks
9 September 2024	–	Hospital admission	–
9 September 2024	Accessory breast cancer	Percutaneous needle biopsy of left axillary mass	Pathology report: non-specific invasive ductal carcinoma (Grade II). Immunohistochemistry: - ER: 90% + (strong positive) - PR: 40% + (strong positive) - HER-2: 0 - Ki-67: hot spot area about 80%+
12 September 2024	Nasal papilloma	Nasal mass biopsy	Pathology report: squamous epithelial papillary lesion of the nasal cavity, no malignant features.
19 September 2024	Accessory breast cancer	Neoadjuvant chemotherapy (TAC regimen)	TAC regimen (Docetaxel 130 mg + pirarubicin 70 mg + cyclophosphamide 850 mg, every 3 weeks (q3w)) administered on: 19 September 2024, 10 October 2024, 31 October 2024, 21 November 2024, 13 December 2024 and 3 January 2025.
11 October 2024	Thyroid cancer	Fine-needle aspiration cytology (FNAC) of the thyroid	Cytology: Bethesda Category V, suspicious for papillary thyroid carcinoma.
21 February 2025	Accessory breast cancer	Letrozole 2.5 mg once daily (qd)	Continuous treatment for at least 5 years.
24 February 2025	Accessory breast cancer	Surgical treatment (nipple-sparing subcutaneous mastectomy with axillary lymph node dissection)	Postoperative pathology: neoadjuvant chemotherapy response grade (Miller-Payne grade) 3; left accessory breast invasive non-special type carcinoma (Grade II), no lymphovascular or perineural invasion; no carcinoma seen in breast tissue; 4/12 lymph nodes positive for metastasis. Immunohistochemistry: HER-2: 0 ER: 20% + PR: 5% + Ki-67: 60%+
24 March 2025	Accessory breast cancer	Postoperative radiotherapy	Radiation therapy to the left breast, chest wall and lymphatic drainage regions: 6 MV X-ray beams, 7 fractions, 95% pCTVcw, 50 Gy/2.0 Gy/25 f; 95% pCTVvm, 50 Gy/2.0 Gy/25 f; 95% pCTVsc, 50 Gy/2.0 Gy/25 f, from 24 March 2025 to 28 April 2025.
4 June 2025	Nasal papilloma	Surgical treatment (endoscopic left nasal lesion excision, left nasal cavity tumor resection, left middle turbinate resection)	Postoperative pathology (Figure 4): squamous epithelial papilloma of nasal cavity with chronic inflammation, no malignancy.
3 September 2025	Thyroid cancer	Surgical treatment (total thyroidectomy, central lymph node dissection)	Postoperative pathology: papillary thyroid carcinoma (max diameter 0.6 cm), 4/7 lymph nodes positive for metastasis.
8 September 2025	Thyroid cancer	Levothyroxine 100 μg once daily (qd)	–
8 September 2025	Accessory breast cancer	Abemaciclib 150 mg twice daily (bid)	Continuous treatment for 2 years.
27 November 2025	Thyroid cancer	Radioiodine-131 therapy	From 27 November 2025 to 2 December 2025
30 December 2025	–	Completed comprehensive treatment	–
31 January 2026	–	Follow-up examinations revealed no signs of tumor recurrence.	–

## Discussion

**Figure 5:**
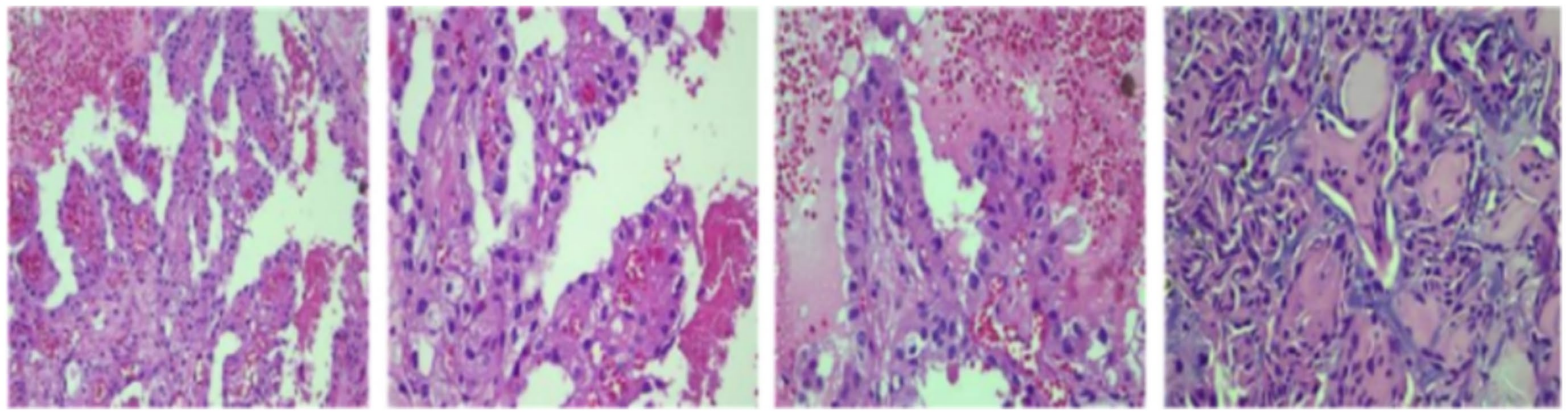
Postoperative pathology of goiter (H&E × 400).

The patient was diagnosed with three primary tumors: an accessory breast carcinoma, a thyroid carcinoma, and a nasal papilloma. This constitutes a case of multiple primary tumors, posing a considerably more complex clinical challenge than a single tumor and necessitating a systematic, integrated diagnostic and management approach. The management strategy was guided by the principles of prioritizing the most life-threatening malignancy, optimizing the treatment schedule, and coordinating interventions in a structured sequence. Specifically, therapeutic priority was given to the metastatic accessory breast carcinoma owing to its aggressive behavior and acute survival risk. Interventions for the other tumors were then strategically planned within available treatment windows, enabling the phased management of the benign and low-risk malignant lesions (Figure 5).

## The core contradiction: highly invasive accessory breast cancer

Accessory breast tissue is an ectopic congenital condition occurring in both sexes, with a reported female predominance (male-to-female ratio ~1:5) and an incidence of 1–6%. It is most frequently located in the axilla (approximately 95% of cases), though other sites including the neck, chest wall, groin, and extremities have been documented. Primary carcinoma arising from accessory breast tissue is rare, constituting only 0.3–0.6% of all breast malignancies (see Table 1 for recent case summaries). Clinical diagnosis is often challenging due to its asymptomatic nature, frequent oversight during axillary examination, and misdiagnosis as benign entities like lipoma or inflammation. The diagnosis of accessory breast carcinoma is established by specific criteria which aim to distinguish it from metastatic axillary deposits or carcinoma of the axillary tail of the breast. Key criteria include: (1) anatomical discontinuity with the orthotopic breast tissue; (2) histological identification of carcinoma coexisting with surrounding glandular lobular or ductal structures; (3) presence of normal or differently typed carcinoma in the ipsilateral breast; and (4) exclusion of other malignancies (e.g., skin adnexal tumors) and specific conditions like tuberculous lymphadenitis. In the present case, intraoperative findings confirmed the axillary mass was independent of the normal breast parenchyma. Histopathological examination revealed ductal carcinoma components within glandular tissue, while the ipsilateral breast was free of malignancy. All other diagnostic exclusions were met, thereby definitively establishing the diagnosis of primary accessory breast carcinoma (3).

The management of accessory breast cancer parallels that of primary breast cancer, involving a multimodal approach that integrates surgery with neoadjuvant or adjuvant chemotherapy, radiotherapy, and endocrine therapy as indicated. The optimal surgical strategy, however, is not standardized and remains a subject of debate. While some authorities recommend radical or modified radical mastectomy of the ipsilateral breast, wide local excision of the tumor with regional lymph node evaluation is often considered the procedure of choice. Axillary lymph node dissection (ALND) is typically performed in the setting of confirmed nodal metastasis. Contrary to this selective approach, Nguyen et al. advocate for the routine inclusion of ALND in the surgical management of accessory breast cancer, irrespective of clinical nodal status (4). This recommendation is based on the anatomical proximity of accessory breast tissue to the axillary nodal basin, which is postulated to increase the propensity for lymphatic invasion and metastasis (5).

The immunohistochemical profile of this accessory breast cancer was consistent with a Luminal B subtype (HR+/HER2-, Ki-67 index of 80%), indicative of high proliferative activity and an increased risk of recurrence and metastasis. The presence of axillary lymph node metastasis represented the most immediate threat. The patient exhibited a partial response to six cycles of neoadjuvant TAC chemotherapy, with the left axillary mass reducing by over 50% prior to surgery. Consequently, on February 24, 2025, the patient underwent a prophylactic ipsilateral nipple-sparing subcutaneous mastectomy with axillary lymph node dissection; given the preoperative diagnostic uncertainty and the patient’s preference for risk reduction, this approach was chosen over wide local excision of the accessory lesion alone. In light of the hormone receptor-positive status, the postoperative comprehensive adjuvant regimen included endocrine therapy (an aromatase inhibitor combined with a CDK4/6 inhibitor) and locoregional radiotherapy.

## Elective management: papillary thyroid carcinoma

Papillary thyroid carcinoma (PTC) is typically indolent with an excellent prognosis, and therefore does not necessitate immediate intervention. In the context of a concurrent highly invasive breast cancer, its management can be safely deferred. For this patient, a sequential therapeutic strategy was implemented, consisting of elective definitive surgery followed by thyroid-stimulating hormone suppression therapy and adjuvant radioactive iodine (^131^I) ablation. This approach is consistent with standard, risk-adapted management guidelines for PTC, which recommend definitive surgery followed by TSH suppression and selective radioiodine ablation based on cytological confirmation and lesion characteristics (6). The timing of thyroidectomy was strategically planned to avoid the period of chemotherapy-induced immunosuppression, thereby minimizing the risks of surgical infection and compromised wound healing. This sequenced arrangement prevented any interference with the breast cancer treatment while ensuring both malignancies were addressed in a logical and safe order.

## Interval management: nasal papilloma

In this case, the nasal papilloma was identified as a relatively rare entity accounting for only 0.4% of adenomas (7). These tumors are typically slow-growing and asymptomatic but carry a recognized malignant potential, for which complete surgical excision is the primary treatment. The excision of the left nasal cavity mass was strategically scheduled in the interval between the breast and thyroid cancer surgeries. This timing was based on the rationale that performing this relatively minor procedure with a short recovery time would optimize the overall treatment timeline. It allowed for efficient use of the convalescent period following breast cancer treatment, without compromising the subsequent major thyroid surgery. The patient recovered well with no evidence of recurrence. Given the malignant potential of these lesions, regular postoperative follow-up is essential for monitoring recurrence, as recommended in the literature (8).

While synchronous breast and thyroid primary malignancies are recognized in the literature, the simultaneous occurrence of three histologically distinct lesions—accessory breast carcinoma, papillary thyroid carcinoma, and a benign nasal papilloma—appears to be exceptionally rare. To our knowledge, this specific triad has not been described previously. While the patient’s initial response to treatment has been favorable, managing such a constellation of synchronous tumors presents considerable clinical challenges. We remain vigilant regarding the potential cumulative and interactive effects of different treatment modalities, which may not only increase the risk of treatment-related toxicity but also complicate subsequent therapeutic decision-making.

Compared with previously reported cases of accessory breast cancer—which typically focus on isolated surgical management 9—the present case is distinguished by the need to navigate three concurrent diagnoses. While synchronous breast and thyroid primary malignancies are well-documented in epidemiological series (9), the addition of a benign yet surgically addressed sinonasal lesion creates an uncommon sequencing challenge. The phased approach described herein, prioritizing the most biologically aggressive lesion while preserving windows for elective procedures, may serve as a transferable template for similar multi-neoplasia scenarios.

While the biological interplay between endocrine therapy, radiotherapy, and synchronous thyroid neoplasia remains an area of active investigation (10, 11), this single case does not provide evidence of causal interaction.

Although this report does not describe novel technologies, it underscores the enduring value of meticulous clinical reasoning and coordinated multidisciplinary care in navigating complex, multi-site neoplasia.

In summary, patients presenting with multiple primary tumors require a structured, multidisciplinary, and longitudinal follow-up strategy that simultaneously monitors for progression across all involved sites.

## Conclusion

This case establishes two pivotal clinical principles. First, it underscores that primary accessory breast cancer must be a top differential diagnosis in any woman presenting with an isolated axillary mass, irrespective of normal breast imaging. Second, it illustrates a pragmatic, multidisciplinary strategy for managing synchronous tumors in a single patient: prioritize treatment based on oncologic urgency, sequence interventions strategically to minimize therapeutic conflict, and maintain rigorous, integrated long-term surveillance. Adherence to this paradigm can optimize outcomes for patients with complex, multi-neoplastic conditions.

## Data Availability

The original contributions presented in the study are included in the article/supplementary material, further inquiries can be directed to the corresponding author.
